# Current regulatory approaches for accessing potential COVID-19 therapies

**DOI:** 10.1186/s40545-020-00222-6

**Published:** 2020-05-16

**Authors:** Vesa Halimi, Armond Daci, Simona Stojanovska, Irina Panovska-Stavridis, Milena Stevanovic, Venko Filipce, Aleksandra Grozdanova

**Affiliations:** 1 Faculty of Pharmacy, University Ss. Cyril and Methodius, Skopje, North Macedonia; 2 Department of Pharmacy, Faculty of Medicine, University of Prishtina, Prishtina, Kosovo; 3 University Clinic of Hematology, Medical Faculty, University Ss. Cyril and Methodius, Skopje, North Macedonia; 4 University Clinic of Infection diseases and febrile conditions, Medical Faculty, University Ss. Cyril and Methodius, Skopje, North Macedonia; 5 University Clinic for Neurosurgery, Medical Faculty, University Ss. Cyril and Methodius, Skopje, North Macedonia

**Keywords:** COVID-19, Regulatory, EMA, FDA, Clinical studies, Compassionate use, Clinical practice, Off-label use, Emergency use

## Abstract

This commentary aims to elaborate challenges in the regulatory approaches for accessing and investigating COVID-19 potential therapies either with off-label use, compassionate use, emergency use or for clinical trials. Since no therapies have been formally approved and completely effective and safe to date, the best clinical choice is acquired only after consistent and fair communication and collaboration between licensed clinicians, researchers, regulatory authorities, manufacturers and patients.

## Background

The available scientific evidence and previous clinical experiences with SARS - CoV, MERS - CoV and even HIV have urged physicians to consider the use of an array of potential therapies like chloroquine and hydroxychloroquine, remdesivir, lopinavir/ritonavir, interferon beta, monoclonal antibodies, convalescent plasma, hyper immune globulin, antibody-rich blood products either alone or combined with supportive care (e.g., oxygenation, ventilation, fluid management) under several regulatory approaches that healthcare authorities made available. Nevertheless, the use of potential therapies in COVID-19 represents a critical responsibility, considering that these therapies are not approved by competent regulatory authorities to treat this disease, and respectively their safety and efficacy profile is under investigation [1].

### Regulatory landscape for accessing COVID-19 therapies in the EU and US

There are several regulatory approaches for accessing potential therapies in COVID-19 and they can be classified as clinical trials, compassionate use, emergency use and off-label use (Table 1) [1–3].

**Table 1:** Regulatory attributes of clinical trials, compassionate use, emergency use and off-label use

	Clinical trials	Compassionate use	Emergency use	Off-label use
Regulatory approval	✓	✓	✓	No
Scope	Clinical research	Clinical practice	Clinical practice	Clinical practice
Informed consent	✓	✓	No	N/A^a^
Target population	✓	✓	✓	No
Safety reports	✓	✓	✓	N/A ^a^
Ethical board approval	✓	✓	No	No
Control group	✓	No	No	No
Gathering evidence	Efficacy, Safety	Safety	Safety	N/A^a^
Risk-benefit assessment	Group	Group	Group	On a case-by-case basis

^a^This table used unified regulatory characteristics of the EU and the US. Instead of “yes” we used the” tick symbol”. Also, when a unified criteria was not met we used the not applicable (N/A) choice

The European Medicines Agency (EMA), even in this pandemic crisis, remained neutral by leaving within the remit of national regulatory authorities to launch their pragmatic regulatory pathways. Even though the EMA provided scientific advice for national regulatory agencies and manufacturers [3], many countries in Europe launched different regulatory approaches and protocols for accessing potential medicines [4–7]. Moreover, the dosing regimen in the protocols even for off-label use is not the same between countries, not to mention other programs. Under ideal conditions, the off-label program would constitute in the creation of a target patient population, informed consent and track and follow-up reports [8, 9]. Still, prescribing an already approved medicine either for an indication, a dose or a way that is not approved for COVID-19 seems to be very challenging for clinicians. Therefore, under the COVID-19 emergency conditions, it is hard to believe that the off-label use would result to be the best approach for accessing potential medicines, considering the ongoing regulatory debates and the difficulties in assessing risk-benefit for each patient due to the pressurized and stressful situation [8, 9].

Unlike EMA and some European countries, the Food and Drug Administration (FDA) was not very keen toward off-label use, by initiating the approval of the compassionate use, followed by the approval of emergency use for particular treatments and clinical trials [10]. Although terminology and modalities may not be identical, compassionate use programs demanding regulatory approval, informed consent and follow-up information are established in most countries [11, 12], and can be used to facilitate the access of seriously ill COVID-19 patients that cannot have access in clinical studies. Even though the compassionate use program is outlined within the framework of clinical practice, and does not have a control group, it can determine preliminarily safety and efficacy data until a level, within a well-formulated study design and hypothesis. Moreover, compassionate use might be seen as a treatment option for small countries which rarely have access to international clinical studies. In the past, during the interval of 5 years (1984–1989), the unapproved Ganciclovir was prescribed under the compassionate use for treatment of pneumonia Cytomegalovirus (CMV), retinitis CMV and colitis CMV for seriously ill immune-compromised patients and even today after 30 years it remains the preferred therapy for the treatment of CMV [13].

On the other hand, the idea for approving the emergency use relies not just in the emergency circumstances but also in providing legal protection for healthcare professionals and manufacturers for eventual adverse events and medication errors that the potential medicine may cause, as well as prescribing and dispensing a donated medicine free of charge within the framework of the hospital, and not obtaining informed consent for patients while tracking and reporting the treatment’s outcomes [14, 15]. Having regard to the fact that the manufacturer Gilead was called from the licensed clinicians to provide Remdesivir to hospitalized COVID-19 patients under the compassionate use, since 25th January 2020, and based on the methodological issues found at Grein et al.’s paper [16], it remains doubtful whether Gilead or regulatory authorities were not vulnerable toward this program. Even though the regulatory authorities usually approve most of the compassionate use requests when meeting the criteria, the key player in this program is the manufacturer, who is not legally obliged to provide the medicine to patients and is usually reluctant due to several factors (1) the outcomes of the compassionate program are not considered as an evidence for approval; (2) they may have limited quantities of the potential medicine already involved in clinical trials; (3) they may think they will be prejudiced if the medicine is proven to be ineffective and unsafe; (4) they want to bypass the regulatory requirements to provide follow-up information; (5) and the reimbursements issues in some countries [17–19]. According to regulatory authorities, the golden criterion for accessing and investigating potential medicines are clinical trials. The speed and volume of various clinical trials like Discovery, Recovery, Anger, Solidarity, New York Trial, characterized with different study designs, experimental arms, outcome measures, eligibility criteria and hypothesis emphasize the urge to produce significant safety and efficacy data in the middle of this pandemic crisis [20, 21]. The golden criterion for the elucidation of the safety and efficacy profile of the potential therapies in COVID-19 does not rely just on the pursuit of randomized clinical studies under an ideal sample size and power, but one should analyze carefully and considerate the other aspects of the study design (e.g. study duration and masking), eligibility criteria (enrolling participants with similar characteristics) and clinically meaningful endpoints.

## Conclusion

Having regard to the diverse clinical presentation, course and progression of COVID-19 and the limited medical supply, regulatory authorities should establish robust regulatory programs that minimize clinical dilemmas in choosing the best therapy for COVID-19 patients. While clinicians should continue to inform COVID-19 patients or their legal representatives for the potential outcomes of therapies, obtaining informed consent from them is crucial for facilitating the clinical decision. Based on the available data, we believe that in COVID-19, no golden criterion is met in clinical research, not to mention clinical practice. Therefore, compassionate use program’s mechanism with no control group might be shifted from “outside of clinical studies” to “parallel with clinical studies” to permit clinical studies to be implemented within their rigorous and narrow framework. However, how safe, effective and correct these current regulatory approaches have been, remains to be verified in the future.

## Data Availability

Vesa Halimi, Armond Daci, Simona Stojanovska, Irina Panovska- Stavridis, Milena Stevanovic, and Venko Filipce, Aleksandra Grozdanova, hereby declare that this option is not applicable.

## Abbreviations

EMA: European Medicines Agency
FDA: Food and Drug Administration
CMV: Cytomegalovirus
SARS - CoV-2: Severe Acute Respiratory Syndrome Coronavirus 2
SARS - CoV: Severe Acute Respiratory Syndrome Coronavirus
MERS – CoV: Middle East Respiratory Syndrome Coronavirus
HIV: Human Immunodeficiency Virus
